# Protein–Peptide Docking with ESMFold Language Model

**DOI:** 10.1021/acs.jctc.4c01585

**Published:** 2025-03-07

**Authors:** Mateusz Zalewski, Björn Wallner, Sebastian Kmiecik

**Affiliations:** † Biological and Chemical Research Center, Faculty of Chemistry, University of Warsaw, Pasteura 1, 02-093 Warsaw, Poland; ‡ Department of Physics, Chemistry and Biology, Linköping University, Linköping 58 183, Sweden

## Abstract

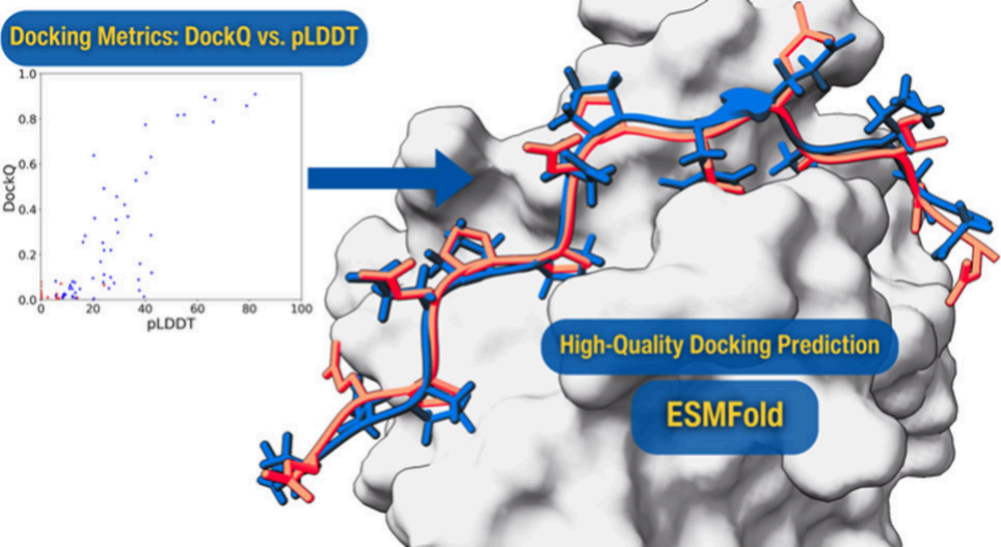

Designing peptide therapeutics requires precise peptide docking, which remains a challenge. We assessed the ESMFold language model, originally designed for protein structure prediction, for its effectiveness in protein–peptide docking. Various docking strategies, including polyglycine linkers and sampling-enhancing modifications, were explored. The number of acceptable-quality models among top-ranking results is comparable to traditional methods and generally lower than AlphaFold-Multimer or Alphafold 3, though ESMFold surpasses it in some cases. The combination of result quality and computational efficiency underscores ESMFold’s potential value as a component in a consensus approach for high-throughput peptide design.

## Introduction

Protein–peptide interactions are essential to many biological processes, or can play a key role when designed as peptide therapeutics. A comprehensive understanding of these interactions is crucial for unraveling disease mechanisms and guiding the rational design of therapeutics. Peptides, with their high specificity, selectivity, and favorable safety profiles, stand out as unique therapeutic candidates compared to small molecules and antibodies due to their natural origin and minimal toxicity.^1^

While traditional experimental studies of protein–peptide complexes using X-ray crystallography and NMR spectroscopy are insightful, they are also expensive, time-consuming, and face scalability issues.^2^ These challenges have spurred efforts toward computational methods in rational drug design, which leverage computer modeling to understand and predict protein structures and interactions.^3^ Despite the complexities presented by the flexible and disordered nature of peptides, these computational techniques are increasingly important for drug development.

In recent years, structural bioinformatics has been transformed by AlphaFold2 (AF2), an AI tool developed by DeepMind that achieved near-experimental accuracy in protein structure prediction.^4^ AF2’s applications soon expanded to include protein–peptide interaction modeling. Initially, this involved using a monomeric version of AF2 with a polyglycine linker to connect peptides and proteins, effectively modeling peptide–protein complexes.^5^ Later efforts shifted to AlphaFold-Multimer (AFM), an AF2 extension trained on protein complexes.^6^ AFM docking, including forced sampling,^7^ achieved 59% accuracy in protein–peptide docking, outperforming traditional methods like ZDOCK,^8^ CABS-dock,^9^ PIPER-FlexPepDock,^10^ and InterPep2.^11^ When benchmarked against other methods, AFM showed 53% accuracy, which increased to 60% when combined with AutoDockCrankPep,^12^ highlighting the potential of hybrid approaches for improving docking predictions and enhancing the study of protein–peptide interactions.

Following the strides made by AlphaFold, ESMFold emerged as another robust tool for protein structure prediction, utilizing a distinct approach.^13^ Unlike AlphaFold, which relies heavily on extensive databases of protein structures and sequence alignments, ESMFold employs embeddings from protein language models (pLMs) derived from vast sequences. This method allows ESMFold to excel particularly in scenarios where limited structural data exists, as it captures more generalized sequence features and patterns through its advanced language modeling techniques. This shift from reliance on direct structural analogs to leveraging learned sequence contexts enables ESMFold to offer unique advantages in predicting novel or less-characterized protein structures.

To our knowledge, ESMFold has not yet been tested specifically for protein–peptide docking. In this work, we investigate the potential of the ESMFold language model for this application and compare its performance with AlphaFold-Multimer with an improved sampling protocol^7^ and AlphaFold 3.^14^

## Materials and Methods

### Dataset

In this study, to evaluate ESMFold’s performance in predicting protein–peptide interactions and to compare it with AlphaFold-Multimer (AFM) augmented by an extended sampling approach,^7^ we used the dataset created and described by Wallner et al.^7^ This dataset was assembled by selecting one representative structure per ECOD (Evolutionary Classification of Protein Domains)^15^ family from the test set in Lei et al.^16^ Referred to here as Dataset 1, it comprises 112 experimental protein–peptide complex structures obtained from the PDB^17^ (see Supplementary Table 1). The 6UEB structure was excluded due to memory constraints in the Colab environment.

Additionally, to compare our results with AlphaFold 3 (AF3) and other AlphaFold-based protocols, we used the dataset introduced by Manshour et al.^14^ Referred to here as Dataset 2, this benchmark consists of 60 protein–peptide structures deposited in the PDB after the AF3 training set cutoff date. Details are provided in the Supporting Information in section “Dataset 2”.

### ESMFold

ESMFold is a deep-learning method that uses the ESM-2 protein language model to predict protein structures directly from amino acid sequences. Unlike AF2, ESMFold does not rely on external databases, template searches, or Multiple Sequence Alignments (MSA). It provides accuracy and resolution comparable to AlphaFold while being up to 60 times faster, depending on the sequence length.^13^ In this study, we used the version of ESMFold implemented in ColabFold notebook.^18^ Since ESMFold is designed for single-chain predictions, we performed protein–peptide docking by introducing a flexible linker (a polyglycine chain) between amino acid chains, similar to early AF2 applications.^5^ This linker was removed from the model after prediction.

### Evaluation Metrics

To evaluate docking success, we used the DockQ score,^19^ which measures protein–peptide docking quality. The DockQ score, ranging from 0 to 1, combines CAPRI criteria:^20^ LRMSD, IRMSD, and FNAT. Scores between 0.23 and 0.5 are considered acceptable, 0.5 to 0.8 medium quality, and 0.8 or above high quality.

We assessed prediction confidence and reliability using the predicted Local Distance Difference Test (pLDDT) value from ESMFold. While pLDDT accuracy can vary based on the protein’s characteristics, ESMFold predictions often reach experimental-like accuracy for high-confidence predictions.^13^ There is also a high correlation between pLDDT scores from ESMFold and AlphaFold.^13^ A prediction is considered high-confidence if the mean pLDDT value exceeds 70.^13^

## Results and Discussion

This study aimed to assess the viability of using ESMFold for molecular docking. It involves inserting a polyglycine linker between the receptor and peptide sequences to facilitate multichain structure predictions. In this work, we tested various modeling variants and reported the quality of the top-ranked models. The output from ESMFold was either a single model, or, if multiple models were available, we applied a scoring scheme based on the pLDDT confidence scores.

### ESMFold Default Protocol

**Figure 1:**
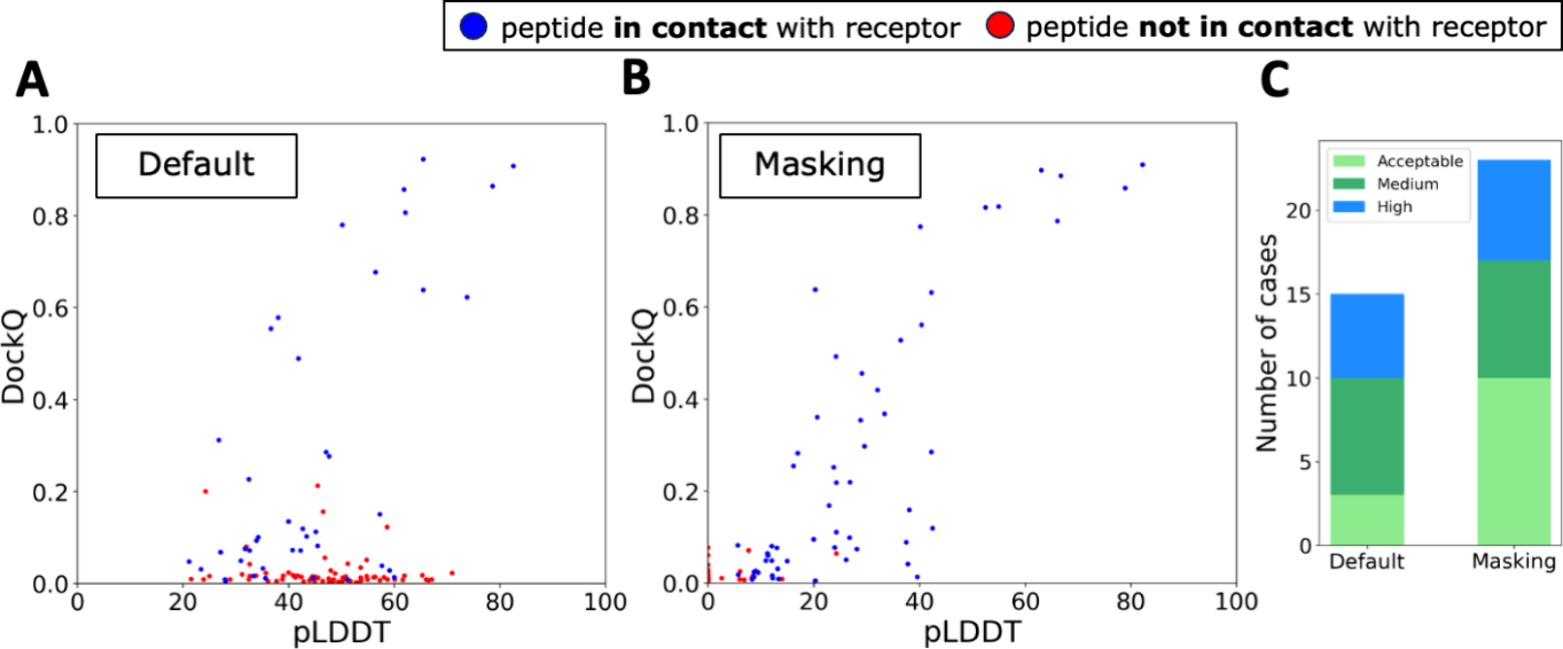
ESMFold docking results with default settings and enhanced sampling variants. (A, B) DockQ vs pLDDT scatter plots for different ESMFold simulation variants: (A) default settings and (B) using a masking approach. Blue dots indicate peptides in contact with the receptor (within 8 Å), while red dots indicate those not in contact (over 8 Å). pLDDT values are the average for each peptide, with (B) showing the weighted mean pLDDT for the top-ranked model out of eight generated per complex. (C) Distribution of high-quality (DockQ ≥ 0.8), medium-quality (0.5 ≤ DockQ < 0.8), and acceptable (0.23 ≤ DockQ < 0.5) docking models for different ESMFold simulation approaches (default, random masking) across 111 complexes, with colors indicating quality.

The initial run, performed using a 30-amino-acid polyglycine linker and default settings, produced a limited number of high-quality structures. Out of 111 cases, 15 structures met or exceeded the acceptable threshold with DockQ scores of ≥0.23, among which only 5 were classified as high quality, as shown in Figure 1A. This figure presents a scatter plot that illustrates the relationship between DockQ scores and pLDDT values.

Many instances placed the peptide more than 8 Å away from the receptor, indicating incorrect docking. These cases were relatively straightforward to identify and exclude. After discarding these, we retained 42 models. This results in an effective success rate of 36% for the viable models: 5 high quality, 7 medium quality, and 3 acceptable.

It is also important to note that predicting a protein–peptide complex of 252 residues (excluding the linker), which is the median length in our dataset, requires only 21 s on an A100 GPU when using a Colab notebook. Additionally, there is an initial setup time of approximately 3 min to install ESMFold on the Colab notebook, but this setup is required only once per session.

### Random Masking Approach

With default settings, a significant number of models failed to position the peptide in direct contact with the receptor (Figure 1A). To address this, we expanded our efforts to generate a larger pool of models. Despite the use of different seed values, ESMFold consistently produced nearly identical output models for each run, which highlighted the need for a strategy to enhance diversity in the generated structures. We adopted a random masking strategy^21^ that involves masking random residues within the input sequence. This approach, using a masking rate of 0.25 and generating 8 structures for each of the 111 complexes, encouraged the production of diverse structural predictions. This method yielded 27 acceptable or better structures, with 6 classified as high-quality from the best of the 8 generated per complex.

Analysis showed the greatest improvement in cases with a low but nonzero initial DockQ score (see Supplementary Figure 1). The challenge, however, lay in selecting the best structure among the 8 generated in each run. We experimented with various scoring strategies based on pLDDT values. Selecting structures with the lowest average pLDDT values across all peptide residues resulted in 20 structures being acceptable or better, including 5 high-quality ones (see Supplementary Figure 2B). A more targeted approach, focusing only on residues within 5 Å of the receptor, produced fewer but more relevant structures, with 2 being acceptable or better, and 5 high-quality (see Supplementary Figure 2C). The most effective method involved weighting the pLDDT scores by the proportion of residues in contact with the receptor, assigning a score of zero to noncontacting residues (see Supplementary Figure 2A). This strategy yielded 23 acceptable or better structures, with 6 high-quality (see Figure 1B and Supplementary Figure 2D).

After discarding cases where the peptide was clearly misdocked (more than 8 Å away from the receptor surface), 57 cases remained. This results in an effective success rate of 40% for the viable models: 6 high-quality, 7 medium-quality, and 10 acceptable structures.

Performing random masking to generate 8 structures for a median dataset protein–peptide complex of 252 residues takes about 61 s on an A100 GPU, a significant increase from the 21 s required for a default run but still efficient for the task. It is important to remember that an additional setup time of about 3 min is required for installing ESMFold on a Colab notebook, but this installation is necessary only once per session.

### Testing Adaptive Recycling and Various Linker Configurations

Apart from masking, we explored an alternative strategy to improve sampling. The default simulations utilized 3 recycles; we extended this to a maximum of 12 recycles by initially performing the standard 3 recycles and then continuing only if the peptide remained more than 8 Å from the receptor. This approach resulted in improved outcomes compared to the default settings but was less effective than masking. Detailed results are provided in Supplementary Figure 3.

Additionally, we explored various polyglycine linker configurations to enhance peptide–receptor contact, testing lengths of 30, 100, and 200 residues at different termini with the masking approach. The results indicated that a 30-residue linker at the C-terminus was optimal, as longer or differently placed linkers did not significantly improve docking quality. Description of detailed results is available in Supplementary Figure 4.

### Comparison to Alpha-Fold-Based Tools

**Figure 2:**
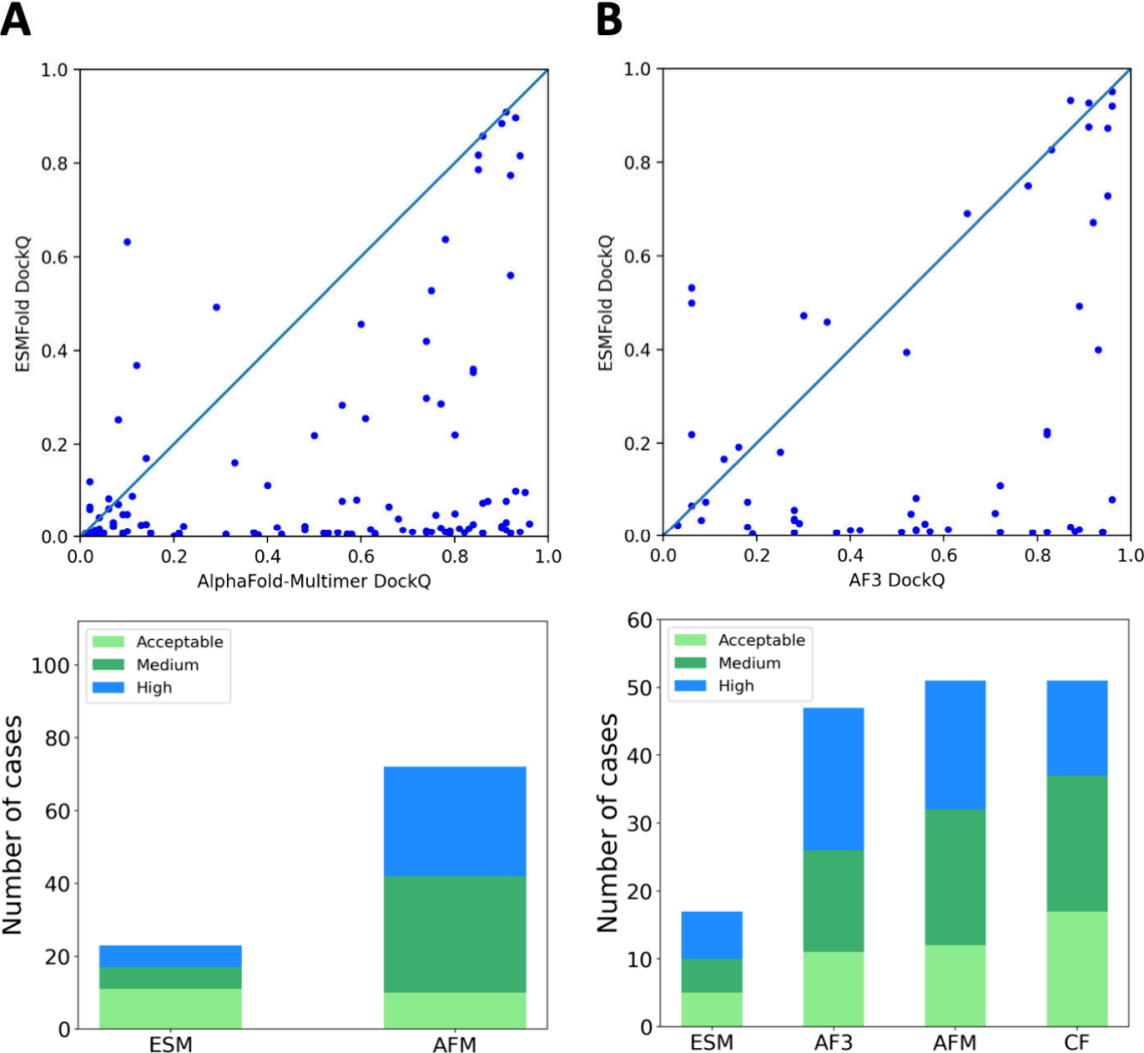
Comparison of ESMFold and AlphaFold-based docking tools on Dataset 1 (A) and Dataset 2 (B). The upper panels show DockQ scatter plots, comparing ESMFold with (A) AlphaFold Multimer (AFM) using enhanced sampling^7^ and (B) AlphaFold 3 (AF3).^14^ The lower panels present the distribution of high (DockQ ≥ 0.8), medium (0.5 ≤ DockQ < 0.8), and acceptable (0.23 ≤ DockQ < 0.5) quality models, comparing ESMFold with (A) AFM with enhanced sampling and (B) AFM, AF3, and ColabFold (CF). Data for AF-based tools are taken from previous studies.^7,14^

**Figure 3:**
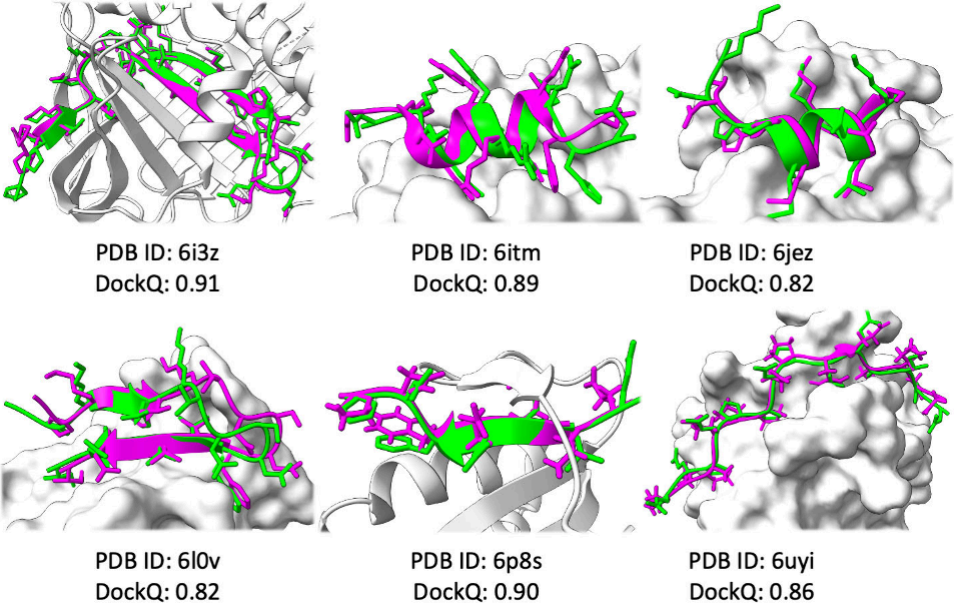
Example high-quality protein–peptide docking results using ESMFold. Protein receptor shown as surface: experimental peptide in magenta; predicted peptide in lime.

For comparisons with AlphaFold-based protocols described in this section, we used the best-performing ESMFold variant (random masking, 30-residue polyglycine linker, weighted pLDDT). First, on Dataset 1, we compared ESMFold to AlphaFold-Multimer with enhanced sampling, which produced 75 acceptable or better models (out of 112), while AlphaFold-Multimer v2.1.0 generated 66.^7^ ESMFold exhibited generally lower overall precision generating 23 acceptable or better structures, including 6 of high quality (see Figure 2A, examples of high-quality models are presented in Figure 3). Notably, ESMfold successfully produced acceptable or better models in three instances where AlphaFold’s predictions were of unacceptable quality (DockQ scores: ESMFold 0.25, 0.63, and 0.37 versus AlphaFold’s 0.08, 0.10, and 0.12; see Figure 2A). Although primarily developed for single-sequence predictions and not trained on multimeric structures or short peptides, ESMFold’s performance here is comparable to non-AI traditional methods^8−11^ (see performance data presented in the work^7^). This demonstrates the potential of evolutionary-scale models to predict biological properties from sequence patterns, suggesting that ESMFold could be further enhanced by including training data on multimeric complexes and short peptides.

Next, to compare our approach with the latest AlphaFold 3 (AF3), we drew upon a recent study by Manshour et al.,^14^ which comprehensively evaluated AlphaFold-Multimer, ColabFold, and AF3 for protein–peptide structure prediction. Using the Manshour et al. benchmark (Dataset 2), ESMFold achieved performance that was comparable to, or even better than, Dataset 1, reaching 28.3% acceptable or better models (Figure 2B) compared to 20.7% in Dataset 1 (Figure 2A). Notably, Manshour et al. found that AlphaFold-based tools yield similar rates of acceptable models overall, although AF3 typically generates a noticeably larger number of high-resolution structures than any other method. Consistent with our findings on Dataset 1, ESMFold also outperformed AF3 in several cases within Dataset 2.

## Conclusions

In this study, we tested various parameters and settings of the ESMFold tool for protein–peptide docking applications. The optimal approach - combining random masking, a 30-residue polyglycine linker, and weighted pLDDT scoring—successfully yielded a significant fraction of acceptable-quality results (about 20.7% for Dataset 1 and 28.3% for Dataset 2, respectively). Although ESMFold did not match the overall accuracy of AlphaFold-based tools, its ability to produce acceptable models—and occasionally outperform AlphaFold-based tools—makes it a valuable component in a consensus-based approach. Furthermore, its speed—about 1 min per run—renders it particularly useful in high-throughput settings. Nevertheless, many of the generated models did not dock properly, underscoring the need for further improvements in ESMFold’s docking accuracy.

In summary, ESMFold’s ability to leverage sequence embeddings and identify crucial binding motifs shows significant promise for protein–peptide docking. Its rapid processing capability is particularly advantageous for applications requiring quick results, such as in peptide-based therapeutic development. Further development could enhance its utility, making it a robust complement to existing methods.

## Data Availability

The input FASTA sequences used for structure prediction are publicly accessible from the Protein Data Bank (PDB) database. All PDB codes corresponding to the dataset are provided in Supporting Information (SI) Table 1. The predictions in this study were performed using the ESMFold model, which is available through ColabFold and can be accessed publicly on https://github.com/sokrypton/ColabFold. Additionally, a modified version of the ESMFold script that includes recycling for enhanced sampling (as described in this paper) as well as all the output structures generated during this study are available on our GitHub repository: https://github.com/ZalewskiMa/ESMFold-docking.

## Supporting Information Available

Additional figures and tables providing detailed analysis of protein–peptide docking with ESMFold. Supplementary Figures 1–5: DockQ distributions, scoring method comparisons, adaptive recycling results, and linker configuration effects. Supplementary Table 1: dataset of protein–peptide complexes used in the study (PDF)
