# Supplementary material for: Protein–Peptide Docking with ESMFold Language Model
Source: J Chem Theory Comput. 2025 Mar 7;21(6):2817–21. doi: 10.1021/acs.jctc.4c01585 (PMC11948316; doi:10.1021/acs.jctc.4c01585)
Supplement: Supplementary file 1 — ct4c01585_si_001.pdf [file ct4c01585_si_001.pdf]

Protein-peptide docking with ESMFold language model

Mateusz Zalewski[a], Björn Wallner[b], Sebastian Kmiecik[a*]

[a] Biological and Chemical Research Center, Faculty of Chemistry, University of Warsaw, Pasteura 1, 02-093 Warsaw, Poland

[b] Department of Physics, Chemistry and Biology, Linköping University, Linköping 58 183, Sweden

*Email: sekmi@chem.uw.edu.pl

Random Masking Approach

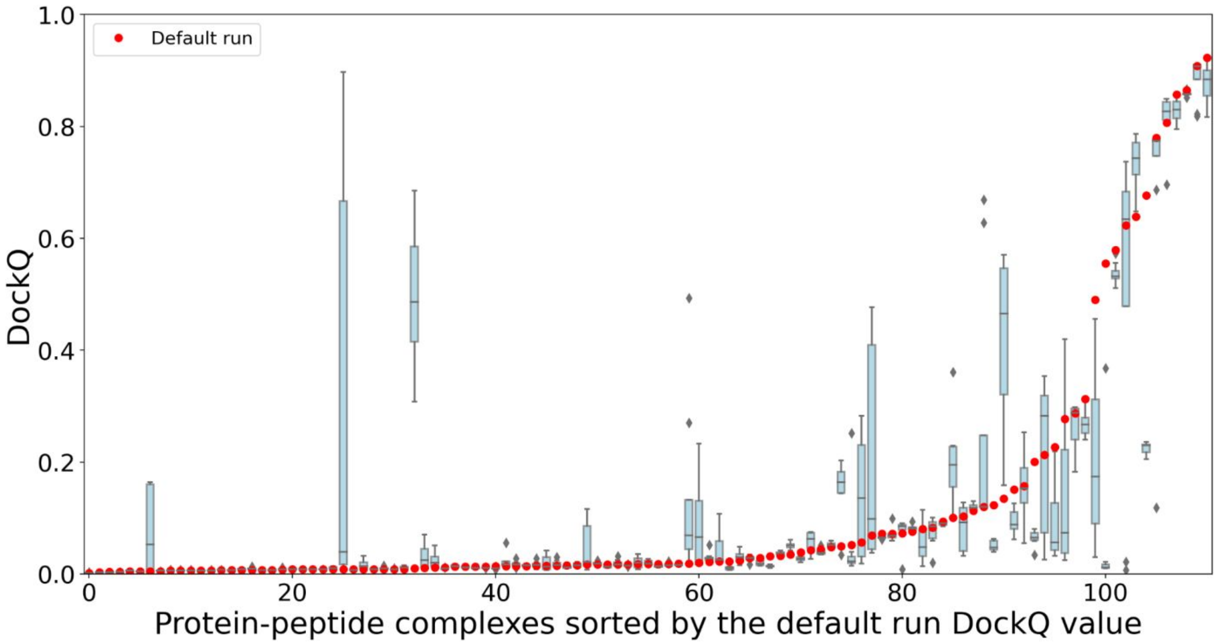

Supplementary Figure 1. Distribution of DockQ values for 111 protein-peptide complexes across 8 different models generated using ESMFold with a masking rate (light blue boxes) and a default run (red dots). Each box represents the interquartile range (IQR) with the median indicated by the horizontal line inside the box, while the whiskers extend to 1.5 times the IQR. Outliers are shown as points outside the whiskers. Protein-peptide complexes are sorted based on the default run DockQ value. The red dots represent the DockQ values from the default run for each complex, providing a comparative baseline against the generated models. The x-axis represents the sorted complexes, labeled every 20th complex, and the y-axis shows the corresponding DockQ values,

indicating the quality of the predicted models. Due to the random masking approach, models with a DockQ value higher than 0 exhibit greater diversity.

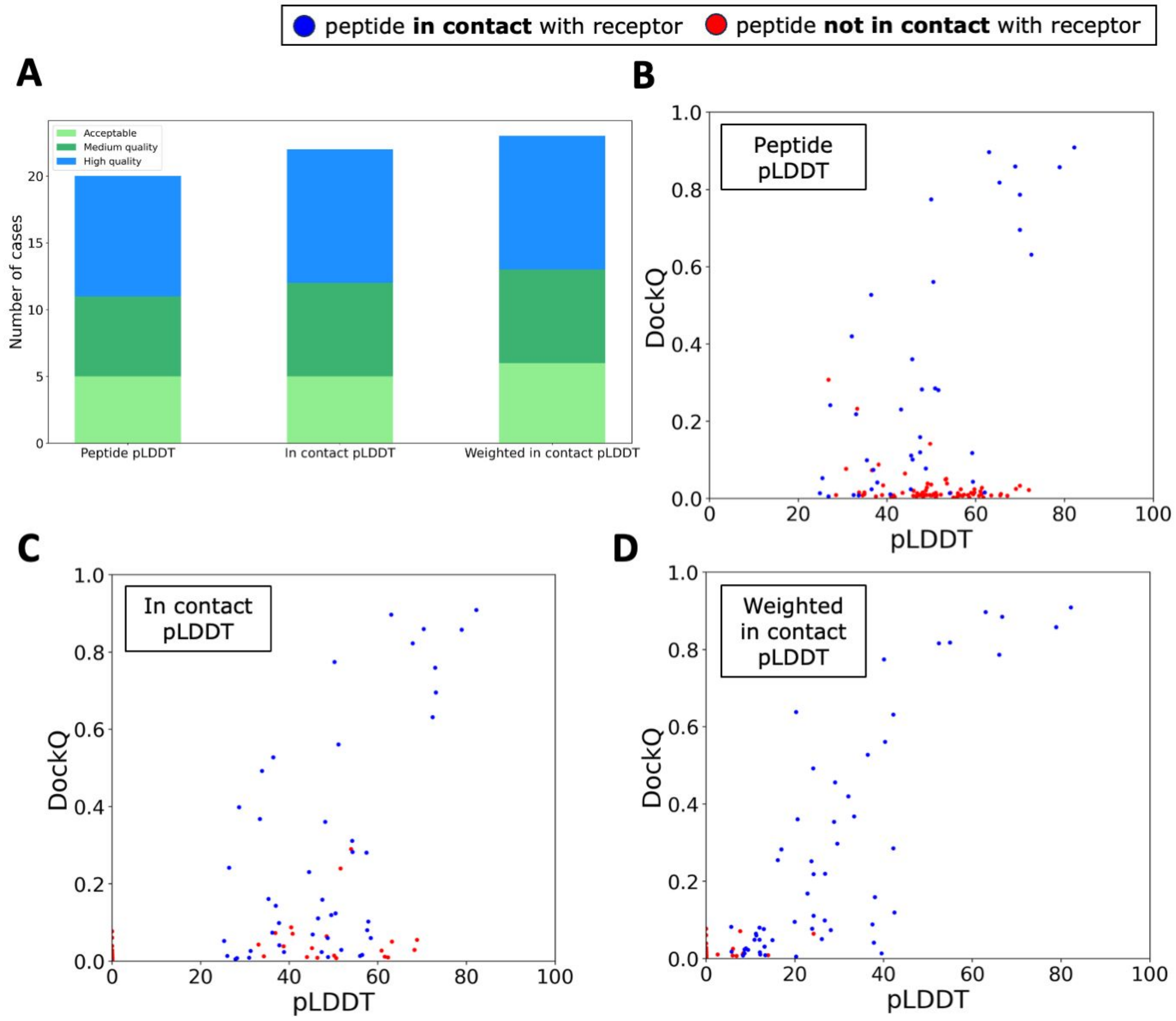

Supplementary Figure 2. ESMFold docking results using different scoring methods.

(A) Bar chart showing the distribution of high-quality (DockQ ≥ 0.8), medium-quality (0.5 ≤ DockQ < 0.8), and acceptable (0.23 ≤ DockQ < 0.5) docking models across ESMFold simulations with different filtering strategy based on 111 complexes. Color coding denotes model quality levels.

(B-D) DockQ vs. pLDDT scatter plots for ESMFold simulations with different filtering strategies: (B) Best peptide pLDDT value, (C) Best pLDDT value calculated for peptide residues in contact with receptor, (D) Best pLDDT value calculated for peptide residues in contact with receptor, weighted by number of residues in contact. Red dots represent peptides with CA atoms located more than 8Å from the CA atoms of the receptor, while blue dots represent peptides within 8Å. Reported pLDDT values are weighted pLDDT values for the top-ranked model out of eight generated per complex.

Testing Adaptive Recycling and Various Linker Configurations

As described in the manuscript, we initially conducted all simulations using the default setting of 3 recycles. Increasing the number of recycles could potentially enhance docking accuracy but also increase computational time, potentially offsetting the speed benefits of ESMFold. To balance these considerations and explore the impact of additional recycles on docking performance, we developed an adaptive approach. This algorithm allowed ESMFold to perform its standard 3 recycles, and then continue with additional recycles only if the peptide remained more than 8Å away from the receptor, up to a maximum of 12 recycles.

Using the 200 poly-glycine linker, this method yielded a total of 18 structures that were acceptable or better, of which 6 were high-quality (Supplementary Figure 3B), providing a slight improvement over the 15 structures that were acceptable or better, of which 5 were high-quality obtained from the default simulation (see Figure 1A). A similar number of acceptable or better structures was achieved using a 30 poly-glycine linker (see Supplementary Figure 3C); however, this variant produced only 5 high-quality structures among the acceptable ones (see Supplementary Figure 3A).

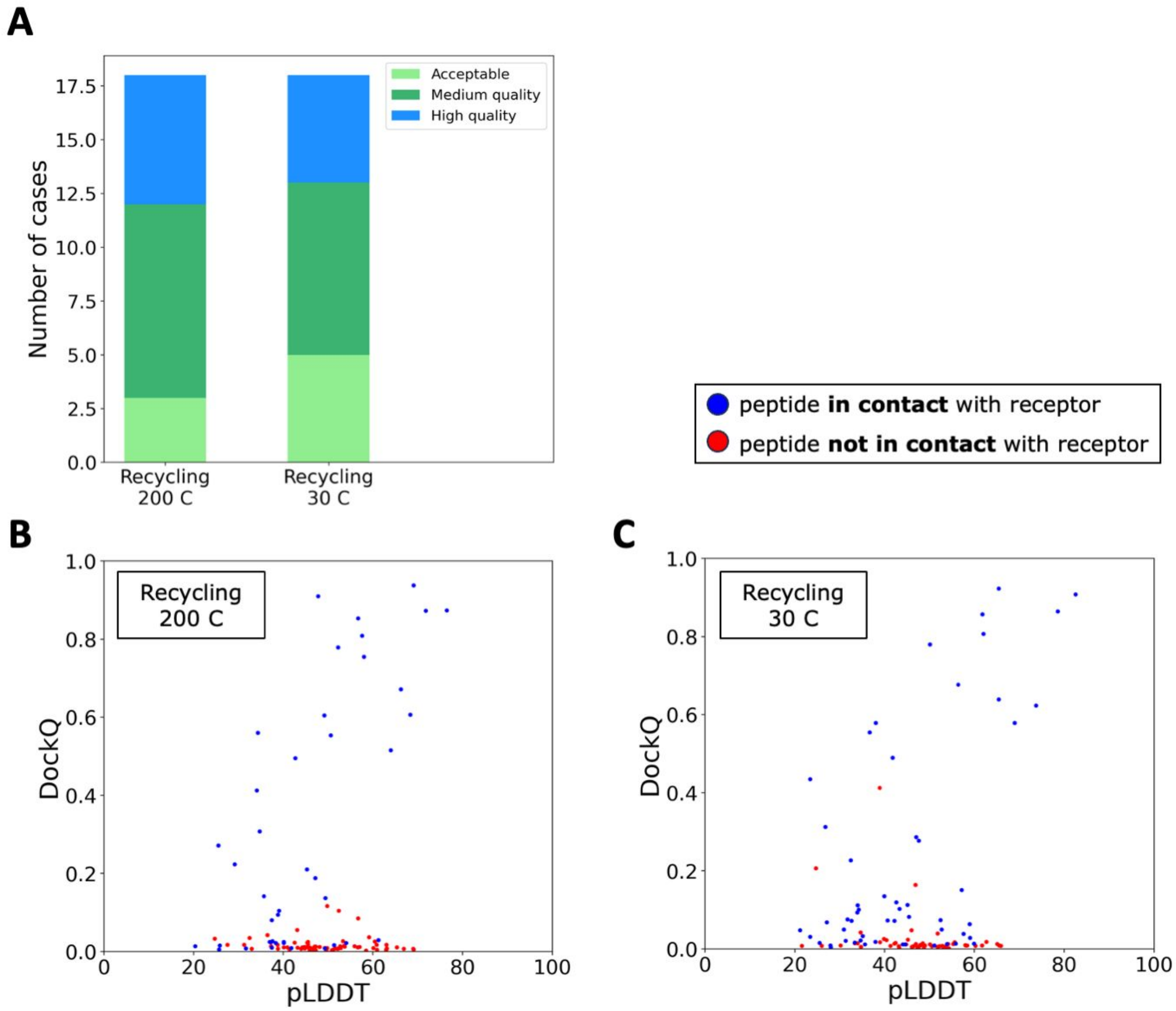

Supplementary Figure 3. ESMFold docking results using a recycling strategy with different linker lengths.

(A) Bar chart showing the distribution of high-quality (DockQ ≥ 0.8), medium-quality (0.5 ≤ DockQ < 0.8), and acceptable (0.23 ≤ DockQ < 0.5) docking models across ESMFold recycling simulations with different linker lengths based on 111 complexes. Color coding denotes model quality levels.

(B-C) DockQ vs. pLDDT scatter plots for ESMFold recycling simulations with different linker length: (B) C-term linker with 200aa length, (C) C-term linker with 30aa length. Red dots represent peptides with CA atoms located more than 8Å from the CA atoms of the receptor, while blue dots represent peptides within 8Å. Reported pLDDT values are weighted pLDDT values for the top-ranked model out of eight generated per complex.

To improve the contact between peptides and receptors, we explored various poly-glycine linker configurations. Initially, a baseline configuration utilized a 30-residue poly-glycine linker at the

C-terminus of the receptor. To assess potential enhancements, we experimented with extending the linker length to 200 residues at both the C-terminus and N-terminus. We also tested a dual-linker configuration with two 100-residue linkers, one at each terminus, to facilitate simultaneous docking of two peptides. All variations employed a random masking strategy with a masking rate of 0.25.

The comparative results from these tests are detailed in Supplementary Figure 4. These findings indicate a distinct advantage for linkers positioned at the C-terminal end of the receptor, suggesting that a 30-residue linker is optimal, as longer linkers did not significantly enhance docking quality. Specifically, using a 200-residue linker at the C-terminal end resulted in 16 structures that were acceptable or better, with 7 being high-quality, based on the best outcome among the 8 generated. Conversely, the N-terminal linker of the same length yielded only 6 acceptable or better structures, of which 2 were high-quality. A similar pattern emerged when docking two peptides simultaneously with 100-residue linkers; the C-terminal linker outperformed the N-terminal, generating 16 acceptable or better structures, including 4 high-quality, compared to only 3 acceptable or better structures, with 1 high-quality, for the N-terminal linker. These outcomes underscore that C-terminal placement of the linker fosters more effective docking. However, neither the 200-residue nor the 100-residue linkers surpassed the performance observed in analogous simulations with 30-residue linkers.

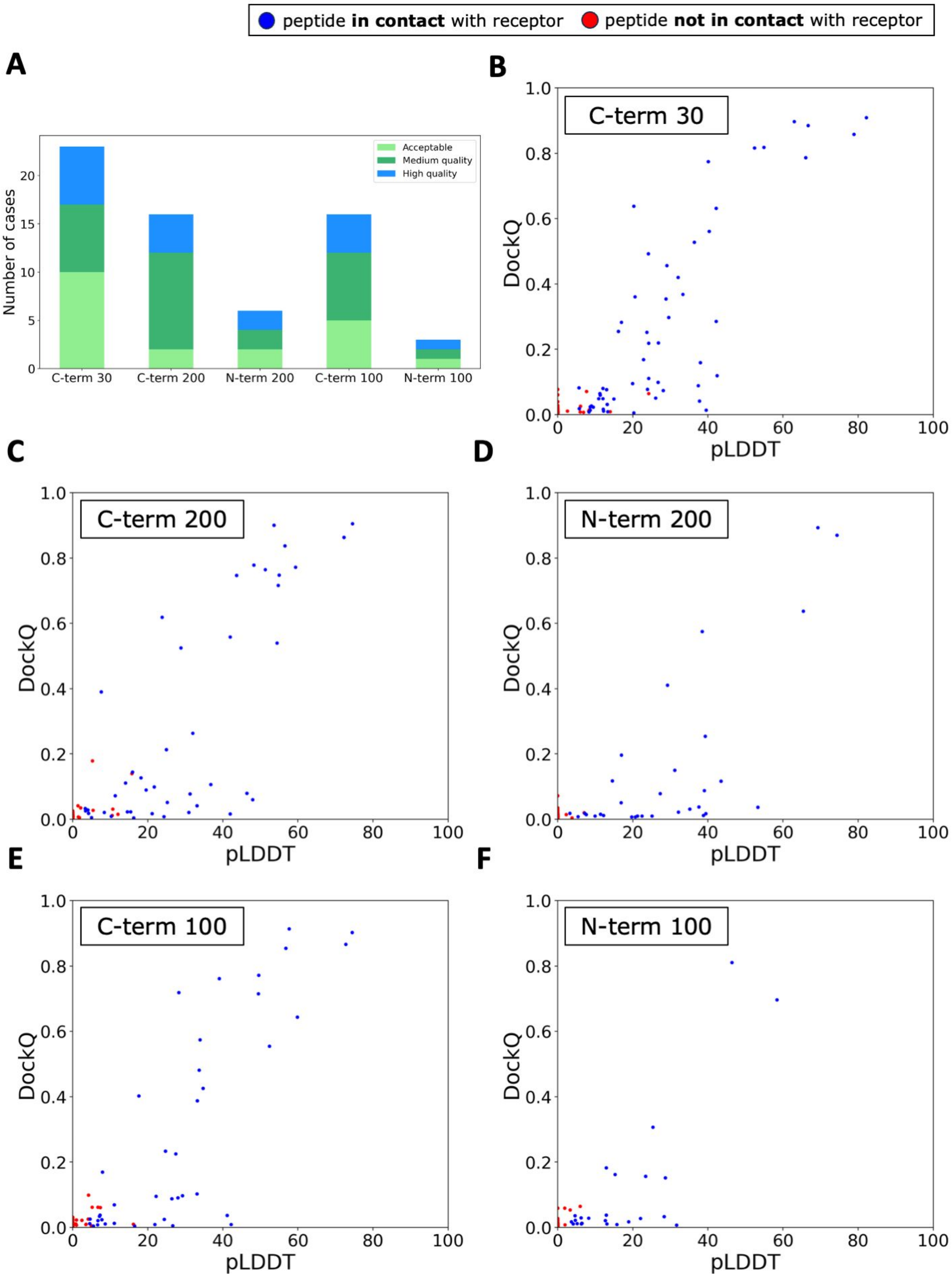

Supplementary Figure 4. ESMFold docking results using different linker variants.

(A) Bar chart showing the distribution of high-quality (DockQ ≥ 0.8), medium-quality (0.5 ≤ DockQ < 0.8), and acceptable (0.23 ≤ DockQ < 0.5) docking models across ESMFold simulations with different linker approaches based on 111 complexes. Color coding denotes model quality levels.

(B-F) DockQ vs. pLDDT scatter plots for ESMFold simulations with different linker variants: (B) C-term linker with 30aa length, (C) C-term linker with 200aa length (D) N-term linker with 200aa length, (E) Double-linker with 100aa length - C-term side, and (F) Double-linker with 100aa length - N-term side. Red dots represent peptides with CA atoms located more than 8Å from the CA atoms of the receptor, while blue dots represent peptides within 8Å. Reported pLDDT values are weighted pLDDT values for the top-ranked model out of eight generated per complex.

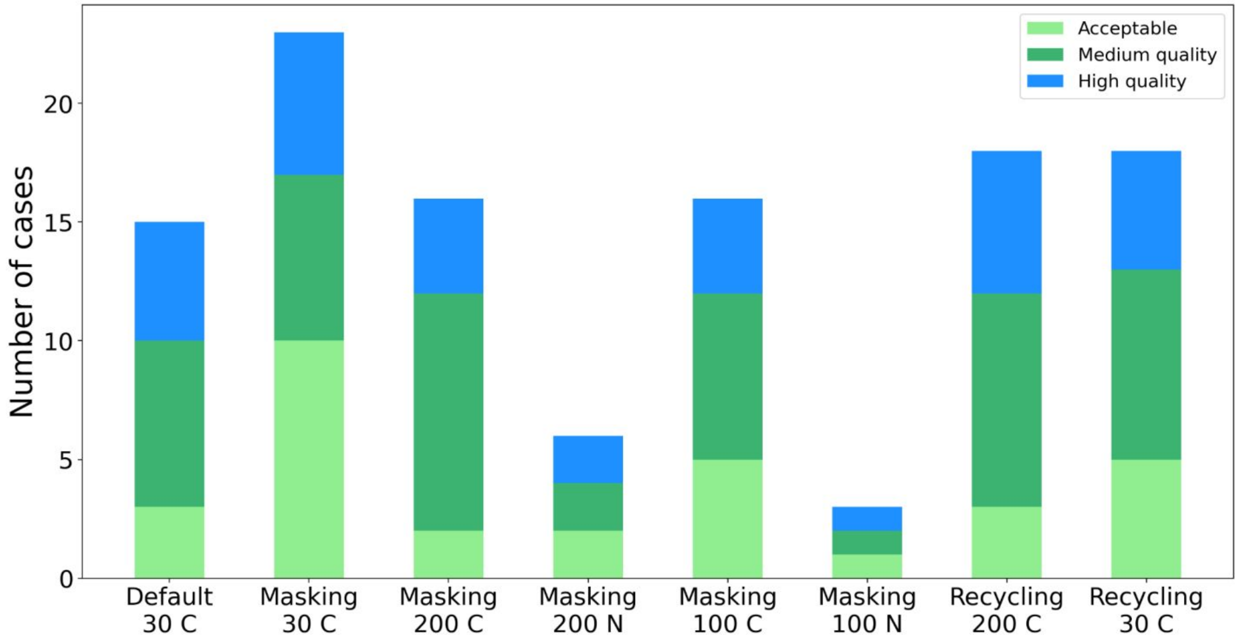

Supplementary Figure 5. Distribution of docking models across various ESMFold simulations variants, categorized by quality levels: high-quality (DockQ ≥ 0.8), medium-quality (0.5 ≤ DockQ < 0.8), and acceptable (0.23 ≤ DockQ < 0.5). The chart also indicates linker length and peptide position (C-terminal or N-terminal), with these features marked by numbers and "C" or "N" symbols.

Dataset 2

This study refers to Dataset 2, which was introduced by Manshour et al[21]. This dataset consists of 60 native protein-peptide structures obtained from the Protein Data Bank (PDB). To ensure a fair evaluation of AF3 and other AF-based methods, only structures deposited after December 1, 2023,

were included in this dataset[21], preventing the use of structures that may have been part of AF3's training set.

For comparison between ESMFold and AF3, we utilized the same ESMFold variant as in Dataset 1. This included employing a random masking approach, incorporating a 30-residue poly-glycine linker to maintain structural flexibility, and applying weighted pLDDT scoring. These methodological choices ensured consistency in evaluating the docking capabilities of both models.

The data presented in Figure 2B of the manuscript for AlphaFold-based methods were kindly provided by Negin Manshour.

Supplementary Table 1. Dataset.

PDB ID	Receptor chain	Peptide chain
6e37	A	B
5qtu	A	H
5qu2	A	D
6a0f	A	C
6a0h	A	C
6e5n	B	A
6e7i	A	P
6ef5	A	P
6gc3	A	B
6hl6	A	S
6hqu	A	I
6hua	A	C
6i3z	A	B
6i42	A	B
6i51	H	I
6i7q	V	H
6i85	A	B
6ifc	A	B
6iiw	A	B
6iqj	A	C
6itm	A	B
6iur	A	C
6j31	A	E
6j8o	B	C
6j8y	C	D
6jez	A	C
6jfa	A	C
6jlh	A	B
6jlj (CY)	C	Y
6jlj (ER)	E	R
6jlj (HX)	H	X
6jnf	A	D

6jwj	A	C
6k61	B	M
6kac (AT)	A	T
6kac (ZV)	Z	V
6kmj	A	C
6kvm	A	C
6l0v	A	B
6l7c	A	S
6l7o	D	Q
6lry	A	B
6mf6	A	C
6msu	A	C
6nid	A	D
6nq3	B	D
6nwe	A	B
6o23	A	E
6o40	A	B
6o51	A	C
6o5o	A	C
6os2	A	B
6oyy	B	A
6p7e	A	U
6p8s	A	E
6p9x	R	P
6pau	A	C
6pbv	A	G
6pek	A	G
6peu	A	M
6pgq	A	B
6pj8	A	B
6pnj	I	M
6ppm	A	E
6pwc	R	B
6q53	A	B

6q68	A	B
6q9f	A	B
6qbb	A	P
6qmp	C	A
6qns	A	S
6qp2	A	C
6qxz	A	B
6r7w	A	B
6r8i	A	B
6rir	A	C
6rko	A	H
6rm8	A	C
6roy	A	C
6rpr	D	G
6rqf	A	G
6rqx	A	B
6s0y	A	C
6s1u	A	I
6s35	A	C
6s6q	A	C
6s7o	A	B
6sa8	A	B
6sat	A	P
6sen	A	L
6slg	A	B
6snc	A	C
6sof	A	F
6spb	F	1
6sw9	7	0
6t1y	A	F
6t2d	A	B
6t7y	A	B
6ttu	D	I
6tyv	A	B

6tzc	B	C
6u39	A	B
6u3i	A	B
6ueb	A	B
6uyi	A	B
6uyo	A	B
6v2h	A	B
6v55	A	Q
6v63	A	Y
6v7o	A	C
6vdb	A	H
6vo5	A	C
